# Leadership Perspectives on Implementing Health Information Exchange: Qualitative Study in a Tertiary Veterans Affairs Medical Center

**DOI:** 10.2196/19249

**Published:** 2021-02-22

**Authors:** Brian E Dixon, Cherie Luckhurst, David A Haggstrom

**Affiliations:** 1 VA HSR&D Center for Health Information and Communication Richard L Roudebush Veterans Affairs Medical Center Indianapolis, IN United States; 2 Department of Epidemiology Indiana University Richard M Fairbanks School of Public Health Indianapolis, IN United States; 3 Center for Biomedical Informatics Regenstrief Institute Indianapolis, IN United States; 4 Orthopaedic Surgery Research Riley Hospital for Children Indiana University School of Medicine Indianapolis, IN United States; 5 Division of General Internal Medicine and Geriatrics Indiana University School of Medicine Indianapolis, IN United States; 6 Center for Health Services Research Regenstrief Institute Indianapolis, IN United States

**Keywords:** health information exchange, veterans, operations research, electronic health record, Indiana, organization

## Abstract

**Background:**

The US Department of Veterans Affairs (VA) seeks to achieve interoperability with other organizations, including non-VA community and regional health information exchanges (HIEs).

**Objective:**

This study aims to understand the perspectives of leaders involved in implementing information exchange between VA and non-VA providers via a community HIE.

**Methods:**

We interviewed operational, clinical, and information technology leaders at one VA facility and its community HIE partner. Respondents discussed their experiences with VA-HIE, including barriers and facilitators to implementation, and the associated impact on health care providers. Transcribed interviews were coded and analyzed using immersion-crystallization methods.

**Results:**

VA and community HIE leaders found training to be a key factor when implementing VA-HIE and worked cooperatively to provide several styles and locations of training. During recruitment, a *high-touch approach* was successfully used to enroll patients and overcome their resistance to opting in. Discussion with leaders revealed the high levels of complexity navigated by VA providers and staff to send and retrieve information. Part of the complexity stemmed from the interconnected web of information systems and human teams necessary to implement VA-HIE information sharing. These interrelationships must be effectively managed to guide organizational decision making.

**Conclusions:**

Organizational leaders perceived information sharing to be of essential value in delivering high-quality, coordinated health care. The VA continues to increase access to outside care through the VA Maintaining Internal Systems and Strengthening Integrated Outside Networks (MISSION) Act. Along with this increase in non-VA medical care, there is a need for greater information sharing between VA and non-VA health care organizations. Insights by leaders into barriers and facilitators to VA-HIE can be applied by other national and regional networks that seek to achieve interoperability across health care delivery systems.

## Introduction

### Background

Health information exchange (HIE) is the electronic transfer of data or information between health care organizations [1], although the format and context of the information may vary. For example, patient information might be used directly in clinical care, or it might inform the management of populations, such as patients attending a clinic for diabetes care [2]. Among US hospitals, 55% reported participating in an organized HIE network that facilitates data sharing [3]. Nonhospital adoption is lower, as 38% of US physicians report sharing information electronically [4].

In 2009, the US Department of Veterans Affairs (VA) launched an interoperability program called Virtual Lifetime Electronic Record Health, which has subsequently been rebranded as the Veterans Health Information Exchange (VHIE) program [5]. The program sought to create a comprehensive, dynamic medical record for each veteran, which is accessible to all VA organizations, regardless of location. Outside the VA, many health care providers are connected to networks that operate within specific geographical regions to exchange health information. To share information, the VA joins with non-VA regional exchanges using the nationwide eHealth Exchange platform [6]. In this paper, HIE efforts between VA and non-VA organizations will be called *VA-HIE*.

Previous research regarding the VA-HIE program has examined the technical infrastructure necessary to enable system interoperability [7-11], the informational value of enabling access to non-VA data [12], the adoption of HIE by patients [13], the impact of HIE on short-term medical costs [14], and the early experiences of clinicians and patients using VA-HIE systems [15]. These studies, like those of the broader US health system, found overall low adoption rates of HIE by both patients and providers. For successful scale-up to a national delivery system that integrates both regional HIE and VA systems, we must identify the implementation practices required for interoperability. Recently, the VA increased its efforts to expand information sharing between its VA medical centers and non-VA community or regional HIEs.

### Objectives

To gain insight into successful interoperability practices, we interviewed organizational leaders associated with a tertiary VA facility following a VA-HIE implementation. The perspectives of VA leaders in information technology, operations, and clinical care and those of community HIE partners were sought to understand the facilitators and barriers to HIE implementation. Insights from leaders within and outside the VA can guide future implementation of HIE in multiple contexts.

## Methods

### Study Design

To understand the factors that serve as facilitators or barriers to interoperability, we conducted semistructured interviews with key leaders associated with VA-HIE implementation at the Richard L. Roudebush Veterans Affairs Medical Center (VAMC) in Indianapolis, Indiana.

### Organizational Setting and Implementation Details

The Indianapolis VA was the organizational setting in which the study was conducted, and the Indiana Health Information Exchange (IHIE) was the community-based HIE network [16] with which VA partnered. Further details of the organizational setting and implementation timeline can be found in Multimedia Appendix 1 [16].

### Participants and Recruitment

We used a purposeful sampling approach to recruit leaders in information technology, operational, and clinical roles within Roudebush VAMC and IHIE. All participants were involved with, or impacted by, VA-HIE implementation. Out of 16 invitations, a total of 12 (75%) leaders responded. In total, 9 individuals from the VA and 3 from the IHIE constituted these organizational categories: information technology (4), operations (6), and clinical care (2).

Specific roles in the information technology category included the VA’s Chief Health Informatics Officer and systems engineers from the HIE community partner and the VA. Operations roles included the VA’s Release of Information (ROI) Officer, Community Coordinator, Director of the Virtual Lifetime Electronic Records Program Office and the CEO of the community HIE partner, and the community partner’s client services representative for the VA. Clinical roles consisted of the VA Chief of Pharmacy and the VA Chief of Ambulatory Services. Informed consent to participate in the study was obtained in writing from all participants.

### Data Collection

Interviews were conducted in person or by phone during the period of June 2014 to August 2016 by investigators DH and BD. Interviews lasted about 45 min (range of 22-62 min) and followed a semistructured format. The interview guide was developed by members of the research team, drawing upon knowledge of HIE, clinical practice, and implementation science.

Interviewers posed 3 main categories of questions regarding VA-HIE implementation: (1) how the informant was involved; (2) what the perceived value of the informant’s organization was; and (3) what barriers and facilitators the informant perceived. (The interview guide, including specific questions and probes, is included in Multimedia Appendix 2.) After listening to initial responses, interviewers probed using open-ended questions and asked for specific examples. The Institutional Review Board at Indiana University and the VA Human Subjects Committee at Roudebush VAMC approved the project.

### Data Analysis

Interviews were audio recorded, transcribed verbatim, deidentified, and checked for accuracy. All investigators independently read each transcript and initially used open coding [17] to capture the essence of the interview. We developed a preliminary coding dictionary using language derived from the data [18]. The dictionary subsequently expanded when new ideas were discovered or when previous codes required finer levels of distinction. Each transcript was coded by at least two investigators.

All investigators, along with a research assistant, met regularly to compare coding and discuss emerging topics. We approached the data inductively using the immersion-crystallization approach to understand each participant’s unique perspective. Immersion-crystallization refers to investigators immersing themselves into the experiences described in the interview transcripts. Crystallization is the emergence of cohesive insights that capture the ideas expressed by multiple transcripts [19]. Data were managed using QSR NVivo 10 software. Analysis was conducted concurrently with data collection, as specified by grounded theory [20], and to gauge when data saturation had been reached.

## Results

### Overview

Overall, we found that leaders from every organizational area were enthusiastic about HIEs and that they were confident that the VA would eventually *get it right*. Given the changes in VA procedures to implement requirements in the Veterans Access, Choice and Accountability Act of 2014, all interviewees imagined a future when the sharing of medical records would be commonplace, accurate, and secure. Many were proud of the VA’s long history of secure information sharing between VA health care organizations across the country. All leaders, including our community HIE partner, imagined that the need for interoperability between information systems (ISs) would continue to increase in the future. From among the leader interview responses, we describe observations across several phases of HIE adoption: recruitment and consent, training, organizational memory, implementation, and sustainability.

### Recruitment and Consent

Recruitment and consent were by far the most frequently discussed topics in our interviews at the VA. Clinical nurses and medical assistants made early efforts at recruitment; however, they had little time to offer detailed explanations of the program to veterans. According to operations leaders, veterans were concerned with how their records would be used, by whom they would be used, and if they would be secure. Eventually, clinical personnel redirected veterans to the ROI office, a division of Health Information Management (HIM) at the VAMC, to learn more about the program. Although clinical staff were relieved of the burden to explain information sharing, recruitment activities increased the administrative load for HIM staff.

All VA leaders with whom we spoke were sensitive to the burden placed on staff by the recruiting and consenting process. Clinical leaders, in particular, noted the impact upon workflow as potentially *disruptive to normal clinical activities* and described clinical personnel as already facing many demands on their time. Likewise, all VA leaders were cognizant of the need for veterans to *opt in* to release their medical information for VA-HIE sharing. In our interviews, operations leaders were aware that the most successful information sharing systems outside of the VA used an *opt-out* approach, where medical patients were automatically enrolled unless they specifically decided not to release their information. In our study, community HIE leaders described the opt-in process as a barrier to greater enrollment.

The VA seeks to protect the medical information of its patients, and thus, veterans were required to expressly ask to join (opt in) the VA-HIE project and agree to the release of their medical records. Operations leaders agreed that the *opt-in* approach was restrictive but that *it would take an act of congress* (literally) to change. One HIM (operations) leader recalled that opting in was a *multistep process* that was *unexpectedly time intensive*. One full-time clerk was hired to process *consent* documents, which on a peak day could exceed 60 documents.

Several operations leaders also described a successful strategy wherein veterans recruited other veterans; specifically, one lead employee in the ROI office was himself or herself a veteran. This employee, called by leaders a *super recruiter*, interacted with veterans as a peer, understanding and addressing their concerns and quelling their suspicions about the use of their medical data. A leader in operations described the consistent success of veterans recruiting veterans:

...I think being a Veteran and telling them that you’re using [the VA-HIE system], I heard this chimed out through the other pilot sites; when some of the Veterans did the recruiting, there’s like a camaraderie.

As the enrollment and consent processes were time intensive, HIM (operations) leaders directed recruiting efforts toward patients who had been seen by outside physicians and were most likely to see the benefit of VA-HIE information sharing. One HIM leader put it this way:

We knew who we should target, who were going to have upcoming appointments, who were going to be seen with the non-VA care providers, so that we could...get the biggest bang for our buck.

HIM (operations) leaders reported that their recruitment rate was among the highest in the country. They attributed this success to finding a process for enrollment that did not burden frontline clinical staff. In addition, allowing a super recruiter to establish a personal connection with each veteran was considered to be essential to the program’s success by these leaders. The *high-touch* approach provided greater ease for veterans and thus higher enrollment.

### Training

Training for the launch of the VA-HIE was handled cooperatively by the community HIE and VA trainers:

- The HIE community partner taught group classes to introduce medical assistants, nurses, and physicians to the VA-HIE program. These personnel were shown how to access non-VA data from the VA’s electronic health record (EHR) system. Community HIE operations leaders were pleased with the willingness of nurses, technicians, and assistants to receive the training, which was offered in a classroom setting.
- The VA Community Coordinator (operations) provided one-on-one instruction to a number of staff members who were viewed by their peers as informal leaders. These leaders would help their groups adopt the VA-HIE program to access non-VA information during clinical visits.
- The VA information technology team members gave demonstrations and answered questions in the clinics and at service-level meetings. They created VA-specific brochures and posters to be displayed in the facility.

The intention, according to one clinical leader, was to reach a *critical mass* of trained users. When that mass was reached and personnel began to incorporate data retrieval into their workflows, then training would emerge organically. He said:

Often, [training] is between personnel in the clinic. So, once you get enough people, some of it will spread, hopefully.

Although staff members were enthusiastic about the training, HIM (operations) leaders described some physicians, advanced practice nurses, and other clinical VA personnel as reluctant to participate in the training and to use the product. VA operations leaders did not aggressively promote training or push product use. They believed that the user interface and information retrieval processes were *still rough*. Leaders thought that promoting a rough product would lead to user disappointment. An operations leader described the concern this way:

If you get a system that doesn’t work well...if the system doesn’t work the first time, the doctors aren’t gonna mess with it again. They might have their assistant do it, they might tell somebody to do it, but the doctors for the main part are not gonna take the time if it doesn’t work right away....So, we were fairly hesitant to go and really push with the doctors.

### Organizational Memory

Operations leaders described some pessimistic responses of clinical staff to the product launch. These leaders speculated that pessimism resulted from disappointing past experiences with other initiatives. They reported that when several software or web-based projects had been launched in the past few years, the promotion of those launches created high expectations. The projects then disappointed early adopters who found them not to be fully developed. One leader told us:

One of the things I have to say, feedback-wise...well, you’re advertising something but it’s not really going to work until about five years from now. I heard that like a broken record, that “we’ve been through this before” and it was hard to get people to help launch and use the product.

The VA has launched many new information technologies and has also been a national leader in quality improvement. Nonetheless, health care providers had mixed experiences with previous roll-outs of health information technology, including early implementation of the personal health record. We found that recent experiences of individuals or even organizational memory were influential on the implementation of the VA-HIE program.

### Implementation and Adoption

From the beginning of the VA-HIE project, leaders were aware that many groups, internal and external to the VAMC, were affected by its implementation. The integration of 2 EHR systems was a complex organizational task. At least two categories of implementation challenges arose: (1) technical (eg, interoperability) and (2) human (eg, coordination among multiple VA groups in different organizational units) challenges.

#### Technical Challenges

Information technology leaders reported that no technical challenges arose that required significant effort or delayed the deployment schedule. One operations leader attributed the success of the technical integration to the experience level of the IHIE, stating:

[The IHIE] was so advanced, and had years of experience doing health exchange—we were really just another on-boarding process for them...

Community HIE leaders also reported a favorable integration process. They described a strong and collegial relationship with VA personnel. Furthermore, because of the success of the technical integration, several community coordinators from other VA sites around the country visited Indianapolis to learn best practices for working with an HIE community partner.

VA operations leaders noted that new personnel championed the adoption of the VA-HIE project. Specifically, those who drove the initiative forward had 5 years or less experience at the VA. New personnel, especially nurses, showed enthusiasm about the project and sought ways to incorporate both recruiting and data extraction activities into their tasks. One leader told us:

It was kind of the younger ones trying to initiate things and push it forward, believing that it was something of value.

Operations leaders noted that nurses and pharmacists were the clinical staff members who most often accessed the VA-HIE system and performed data extraction.

However, VA informatics (information technology) leaders reported some challenges with retrieving data following implementation. In most clinics, plans had not been made to incorporate VA-HIE information retrieval into routine care. Personnel were unsure how to proceed. Many physicians were reluctant to change the clinical workflow. One operations leader recounted a session where he assisted a clinician with data retrieval:

We’d go on [the system] and do a query and if [the system] sat there, we waited for it. [The clinician said], “I like the system, but I’m not going to sit here for five minutes. It didn’t work once. It didn’t work twice. I’ve got to go take care of patients.”

As a result of these early challenges, some HIM operations leaders thought that physicians might underestimate the power and features of the fully functional VA-HIE system. They further speculated that physician adoption might have been higher had the system been fully operational and user friendly when it was launched.

#### Human Challenges

Leaders described clinician uneasiness about relying upon records that were created elsewhere for the purposes of medical decision making. Unknown errors or omissions seemed possible within those outside records. As information sharing between VA and non-VA HIE systems was a relatively new practice, clinical leaders surmised that VA clinicians were concerned about their own liability if medical errors were introduced by inaccurate outside information.

Leaders affirmed the organizational complexity of implementing the VA health care system. They described several different organizational units, system levels, and procedures that were involved in the adoption of VA-HIE information sharing. Interviewees from different departments and settings provided details about their unique perspectives on the implementation process.

In the HIM department, we observed that the process of obtaining patient consent impacted workflows. In addition to explaining the program and answering questions, staff were required to scan the consent forms into the EHR. Medical record staff then recorded the consent in a second system, referred to as the Veteran Authorization Preference system, as well as in a local registry that tracked the number of consents at the facility. Reminders were established in the local registry to re-enroll patients when their current consents expired.

In ambulatory clinics, clinical leaders noted that recruitment tasks competed with existing clinical activities for clinic staff’s time, including physicians, nurses, and practice managers. To streamline the workflow, frontline clinical staff members offered VA-HIE information exchange as a service to the veterans who were seen in the clinic. To retrieve medical records, nurses who had received training would use the VA-HIE system with support from the information technology group.

In the ROI office, veterans who visited were asked by operations staff if they wished to enroll in VA-HIE. The Community Coordinator, in concert with health informatics leadership, used several strategies to obtain the consent of veterans for HIE. Oftentimes, these approaches were opportunistic, such as approaching patients who were waiting in line to pick up prescriptions or receive a flu shot. Veterans could also access their records through the MyHealtheVet (MHV) patient portal and give consent through MHV.

**Figure 1:**
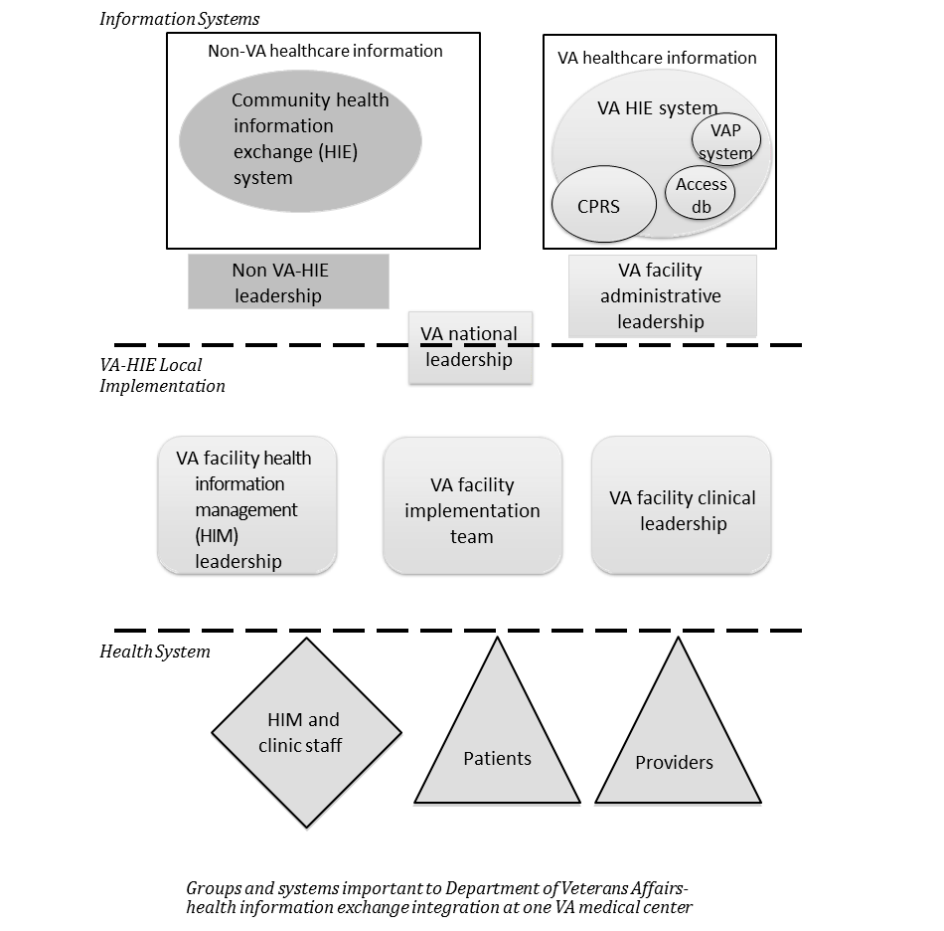
Groups and systems important for integration of VA health information exchange at one VA medical center. Access db: Access database; CPRS: Computerized Patient Record System; HIE: Health Information Exchange; HIM: Health Information Management; VA: US Department of Veterans Affairs; VAP: Veteran Authorization Preference registry.

From our observations, we derived a novel organizational framework (Figure 1). The figure captures the interconnected web of ISs and human teams and the range of staff, provider, and patient stakeholders who were necessary to integrate 2 robust enterprise health ISs. Although the precise configuration of data systems and teams may vary by project, the framework visualizes key groups that were necessary to successfully implement one VA-HIE partnership. This underlying structure can be applied by other national and regional networks that seek to achieve interoperability across health care delivery systems.

In brief, our framework (Figure 1) places consumers of information and the HIM and clinical staff who were in direct contact with the data on the lower level. Information sharing (whether importing patients’ records from outside the VA system or allowing them to be exported from the VA system) was mediated by the facility-based groups in the next layer. (This layer also includes the national offices of the Veteran’s Health Administration who oversaw the VA-HIE demonstration project.) The top level consists of 2 regional systems that exchanged health care information: the VA facility and its community HIE partner. A detailed discussion of the framework is provided in Multimedia Appendix 3. This framework can help identify key organizational units likely to be involved in successful HIE implementation.

### Sustainability

The biggest sustainability challenge from the perspective of community HIE leaders was how to fund the continued integration of the VA and HIE systems. Most community HIEs charge membership fees to participating health organizations. The VA-HIE demonstration project was, by design, a time-limited initiative wherein community HIE fees were no longer supported by the VA after 2 years. Since then, VA leaders have permitted local HIE subscribers to continue to access VA medical records.

Despite financial concerns, community HIE leaders were in agreement about the value of maintaining interoperability between VA and non-VA ISs. They noted a strong, positive response by non-VA organizations to the availability of the VA medical records. One HIE operations leader told us:

I think that even just the mention of the VA to our customers gets their attention—it’s a source of data that people are interested in....So for us, it’s a selling point of our HIE that we have that data....We are glad to have it and we want to continue it.

Sustainability was perceived by VA clinical leaders to rest upon the added value of exchanged information in the clinic and upon the reliability of the information sharing process. In short, the VA-HIE product must perform in the way that it is intended and provide usable results. One operations leader stated:

We need to have a product that’s prime time ready. It’s not because the information isn’t there [now]. It is there but we need to have something that’s clinical, that’s usable in a clinical environment that’s fast, that’s structured appropriately.

A VA information technology leader concurred:

Obviously, it takes a long time—years, decades—whatever, to get to a point that...the system is mature enough to be able to make it work well across all the different data systems.

## Discussion

### Principal Findings

Overall, VA and community HIE leaders believed in the promise of HIE to facilitate high-quality, coordinated care delivery but nonetheless identified several facilitators and barriers to HIE that will need to be addressed for successful, sustainable interoperability between VA and non-VA providers. Increases in veterans’ access to non-VA medical care create a clear need for greater information sharing among VA and non-VA providers. Overall, leaders perceived a strong rationale for integration with non-VA providers to support care coordination and policies that encourage HIE. Organizational leaders also perceived a number of human factors issues that make VA-HIE implementation and workflow challenging from the perspective of frontline providers and staff. These findings provide lessons for other VA sites and other HIE programs throughout the United States and globally.

### Comparison With Previous Work

We observed significant support for the notion by VA and community HIE leaders that VA-HIE, again, is critical to delivering high-quality, coordinated care to veterans. Their support is similar to the findings of Byrne et al [15] who conducted health care provider interviews at other VA-HIE demonstration sites. In this previous study, 96% of veterans felt that VA-HIE would benefit veterans, whereas 71% of VA care providers who used VA-HIE reported positive changes in the care of their patients. Broad support for HIE is likely influenced by policies that create additional incentives to adopt VA-HIE. Community HIE leaders noted that the Veteran’s Choice Act of 2014, which expanded access to non-VA medical care for veterans [21], brought greater demand from non-VA providers to access VA records for veteran patients that may be seen in their practice. Similarly, VA operations leaders noted that demand was also increasing in the other direction, that is, VA providers needed information from non-VA medical records to deliver comprehensive care.

Broader access to non-VA providers will likely increase the chance for fragmentation of care [22] and strengthen the need for VA-HIE [23]. As fragmentation increases in health care delivery, both providers and patients may need to change their mental models from an ownership view of health data to a perspective view that emphasizes continuity of care [24]. To successfully integrate new external health information into clinical work practices, health care providers will need to recognize the potential value of health information from outside their own practice [25,26]. Future research should examine the impact of these policy changes, enabling access to non-VA care for veterans, on the use of VA-HIE and measurable impacts on veterans’ health outcomes.

The original VA policy of intentional consent (opt in) was designed to protect patients and preserve the confidentiality of their records. In contrast, most state and regional health organizations assume patient consent; those patients must opt out to restrict sharing. Knowing this, the majority of leaders whom we interviewed believed that the requirement for veterans to explicitly opt in created the single biggest obstacle to patient adoption of the VA-HIE program. The merits and limitations of opting in versus opting out have been discussed extensively [27]. Previous studies have found that patients are concerned about the possible loss of privacy or misuse of their health data [28,29]. Moreover, patients are concerned about losing control over their health records when HIE systems are implemented [30]. Only a narrow majority (58%) of patients believe that the benefits of sharing health information outweigh the risks [31]. Although these trends are changing in the public sector [32], future research should investigate more fully the veteran response to interoperability.

According to leaders, disappointments around past VA information technology initiatives had a meaningful impact on this demonstration project. In the past, providers may have felt that new information technologies had imposed additional time burdens and were imperfectly executed. Although subjective emotional responses may not be foremost in the planning of informatics product releases, our findings reinforce that such considerations may be very important. Many investigators have described the importance of nontechnological elements in evaluating HIE adoption [33-36]. IS literature suggests that individuals’ feelings about information technology impact their adoption decisions [37]. Furthermore, researchers have suggested that organizational culture may substantially influence both the implementation of new technologies and the continuance of old systems [38]. Health care workflow often is not driven by efficiency alone but by other considerations, such as individual preferences, or organizational and cultural factors that are important to individuals [39,40].

Clinicians were uneasy and concerned about their own liability if medical decisions were based on inaccurate non-VA information. Questions about the medical liability introduced by shared records have been widely discussed [41] and have highlighted the need for accurate matching of patient identities with patient records across systems. Although many of these liability concerns have since been addressed (eg, Office of the National Coordinator for Health Information Technology data brief) [32], new issues have emerged. Local laws continue to create many barriers to information exchange [23]. In 2018, more than 2300 state statutes and regulations were associated with electronic health information and its sharing [42], and these laws have introduced many different barriers to the efficient sharing of health information.

In the VA, federal regulations supportive of interoperability have accompanied expansions in veteran access to community care; however, assembling a critical mass of veterans’ non-VA health records has remained a formidable challenge. Physicians have had little information available about care received outside the VA because the reach of HIE among veterans has been low (4% have consented to data sharing in 2018). Thus, more than 90% of requests from community HIE partners for veteran health data are rejected because of lack of consent on files [26]. Low adoption of HIE is not unique to the VA; recent reviews show that HIEs remain underutilized, integration between systems is not fully developed, and many barriers remain [43-45].

### Current Developments at VA

Our data were collected during a VA-HIE demonstration project, which was implemented at 9 different VA sites and ended after 2 years. The Department of VA continues to make changes that impact information sharing. The Veteran’s Choice Program was replaced in June 2019 by the Veterans Community Care Program, which is similar to Veteran’s Choice but is attached to the VA Maintaining Internal Systems and Strengthening Integrated Outside Networks (MISSION) Act. The VA MISSION Act offers veterans new options for health care by further expanding their eligibility requirements to receive care in the community and increasing resources for in-home care providers and tribal health programs [46]. Most relevant to VA’s information sharing efforts, the act also provides authority to automatically release veteran EHRs to non-VA care providers [47], and thus, veterans no longer need to opt in to share their records. We anticipate that concerns about patient confidentiality may re-emerge in response to the act’s opt-out approach.

Overall, the MISSION Act contains many provisions for improvements in access to health care among veterans. High-quality, coordinated care can be best achieved along with the implementation of an infrastructure of interoperable human-centered technology. The need for an infrastructure that allows access to medical records between organizations—whether public, private, or governmental—has become a national priority [45]. During the COVID pandemic, social isolation has made information sharing even more important, as many clinics and hospitals conduct appointments virtually. Continued examination of both the technical and human challenges of information sharing remains important and timely.

Finally, the VA is currently implementing a new EHR system, a process that will require a decade to fully roll out across all VA facilities. Updating to a modern commercial EHR system will allow VA to make further progress toward its interoperability and HIE goals [48]. The commercial system being implemented is a founding member of the CommonWell Health Alliance, which seeks to establish a nationwide infrastructure for health data exchange [49]. As VA moves forward with its national EHR modernization plan, it should examine the HIE functions afforded through the platform and study how those functions might complement the existing VHIE program. Clinical workflows and technical components differ between the current VA EHR environment and the infrastructure afforded by CommonWell. Understanding these differences and developing approaches that maximize efficiencies for providers and HIM personnel will be critical not only for the success of HIE within the VA but also for the success of its new EHR platform.

### Limitations

We note a few limitations to our study. First, some readers may consider the use of qualitative methods to be a limitation. However, one benefit of a qualitative approach is the real-time capture of dynamic conversations. Interviewers covered a range of topics and were able to dig deeply when warranted. Such fluid revelations of information cannot be achieved when using a static questionnaire. Second, the study was conducted with leaders at one VAMC and its HIE community partner. The IHIE is well established and robust, and thus, the experiences of other VA and HIE leaders may not replicate the partnership described here. Third, each medical center has its own history and culture. VA leaders in our study noted that experience with past VA initiatives might have influenced participation in the VA-HIE project. Other medical centers, having their own history, may respond differently.

### Conclusions

The VA-HIE demonstration project showed how the integration of data across complex networks could be implemented. Leaders at one VAMC and its community partner HIE described the importance of information sharing and its value in providing high-quality patient care. Further discussion with them revealed the daunting levels of complexity VA personnel navigated to send and retrieve information. These VA and HIE leaders discussed the time-intensive process of asking patients to share their medical records. This VA found success in having veterans speak to other veterans, yielding the highest recruitment levels in the country.

Our interviews revealed that the technical compatibility between the 2 systems is not necessarily the major management challenge; rather, it is the coordination of the complex interrelationships among entities within the local and national VA. The synthesis of the observations from organizational leadership responsible for HIE implementation and stakeholders impacted by HIE adoption led us to create a new organizational framework to describe and visualize those relationships. The lessons learned advance implementation science and can be applied by other national and regional networks that seek to achieve interoperability goals across health care delivery systems.

## Appendix

Multimedia Appendix 1 Details of organizational setting and implementation.

Multimedia Appendix 2 Interview guide for Indianapolis VA-HIE evaluation.

Multimedia Appendix 3 Models of entities required to integrate two information systems.

## Abbreviations

EHR: electronic health record
HIE: health information exchange
HIM: Health Information Management
IHIE: Indiana Health Information Exchange
IS: information system
MHV: MyHealtheVet
MISSION: Maintaining Internal Systems and Strengthening Integrated Outside Networks
ROI: release of information
VA: veterans affairs
VAMC: Veterans Affairs Medical Center
VHIE: Veterans Health Information Exchange
