# Supplementary material for: Leadership Perspectives on Implementing Health Information Exchange: Qualitative Study in a Tertiary Veterans Affairs Medical Center
Source: JMIR Med Inform. 2021 Feb 22;9(2):e19249. doi: 10.2196/19249 (PMC7939932; doi:10.2196/19249)
Supplement: Multimedia Appendix 1 [file medinform_v9i2e19249_app1.docx]

### Details of Organizational Setting

The RLR VAMC provides inpatient and outpatient services to more than 60,000 patients annually. Health care services are delivered at a tertiary medical center, as well as three community-based outpatient clinics located in central and southern Indiana.

The Indiana Network for Patient Care (INPC) is one of the largest community-based HIE networks in the U.S. [1]. The INPC is managed by the Indiana Health Information Exchange (IHIE), an Indiana-based not-for-profit corporation that seeks to improve the quality, safety, and efficiency of healthcare in Indiana. The INPC connects more than 120 facilities representing 38 health systems across the state, including hospitals, physicians’ practices, pharmacy networks, long-term post-acute care facilities, laboratories, and radiology centers.

The VA-HIE initiative is a national VHA program that facilitates electronic data exchange between the VA and more than 30 community-based HIE networks, plus the U.S. Department of Defense and other federal agencies. RLR VAMC was the fourth site in the VHA system to implement VA-HIE, and it focuses upon the exchange of health information between RLR VAMC and the INPC.

### Details of Implementation & Timeline

Implementation of VA-HIE at RLR VAMC and the INPC occurred in 2011; October 2011 marked the “go-live” month (the time when VA providers at RLR VAMC were first able to access non-VHA health information in real time). The implementation phases of VA-HIE are shown in Figure A1. It is organized into five phases that are distinct but with overlapping chronologies.

*
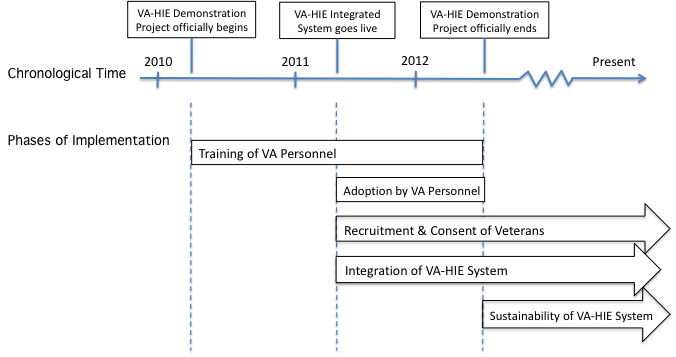
*

*Figure A1.* *Phases of VA-HIE implementation over time.*

From late 2010 through 2012, a VA Community Coordinator was contracted by the national VA program office to facilitate implementation of VA-HIE at RLR VAMC. His duties included conducting training for staff on how to access non-VA data, workflow redesign to incorporate non-VA data, and the development of Veteran consent processes to enable access to non-VA data. Furthermore, the coordinator facilitated technical deployment, tracked implementation progress, and addressed issues with connectivity and the display of non-VA data in VA systems.

As of 2018, the VA-HIE program was deployed nationwide across the VA. RLR VAMC continues to exchange data with the INPC, although the coordination of data exchange is now distributed across many operational roles within RLR VAMC and the national VA program office.

Reference for Appendix A

Appendix A [1] Overhage JM (2016). The Indiana health information exchange. *Health Information Exchange: Navigating and Managing a Network of Health Information Systems, 1*: 267-79.
