# Supplementary material for: Leadership Perspectives on Implementing Health Information Exchange: Qualitative Study in a Tertiary Veterans Affairs Medical Center
Source: JMIR Med Inform. 2021 Feb 22;9(2):e19249. doi: 10.2196/19249 (PMC7939932; doi:10.2196/19249)
Supplement: Multimedia Appendix 2 [file medinform_v9i2e19249_app2.docx]

Interview Guide for Indianapolis VA HIE Evaluation

To be completed prior to interview-

**Organization:**

**Interviewee:**

Role:

(e.g. Medical Center Director)

- Introduce yourself to the interviewee and establish rapport
- Thank the interviewee for their willingness to participate and remind them that you expect the total interview to take no more than 60 minutes.
- Read the statement of purpose and clarify any questions the interviewee may have.
- Tell the interviewee "If you are ready, we will get started "

Interview Questions-

***VLER Participants***

Did you interact directly with individuals at the regional HIE partner(s)? (IHIE & participating organizations)

- IF YES Please describe your interactions with the regional HIE partner(s).

Probes:

- What barriers or challenges did you encounter to collaborating or interacting?
- With regard to your interactions, what has been working well?
- Did your facility seen any change in outcomes as a result of participation in VLER?

| **Possible Outcomes to Ask About** |
| --- |
| Health care Quality/Patient Safety |
| Administrative costs |
| Costs of Care |
| Re-admissions or service utilization |

- Did you monitor VLER activity within the facility? If so, how?

Probes:

- What metrics did you use to monitor VLER activity? (May want to probe here, this could be a hard question to answer and you may have interest in specific sorts of metrics.)

***VA Local Site Leaders***

- What aspects of the VLER implementation and rollout worked well for your facility?
- What aspects of the VLER implementation and rollout could have been better? Why?

Probes:

- How did you decide what specific populations of patients to target?
- What methods did you use to reach out to veterans?
- How did you educate VA clinicians and staff about VLER and the availability of external data through VistaWeb? {Open Ended}

Probes:

- How did that education go? What challenges did you encounter?
- What resources for VLER were provided by your facility? On what aspects of the project did they focus?
- What were the major barriers or challenges associated with VLER implementation?

| Barriers to Ask About |
| --- |
| Human Resources - IT Personnel, User training, project management |
| Privacy and Security - Breeches occurring in external organizations, impact on release of information processes within the VA |
| Reliability of Information from Outside Organizations |
| Timeliness of External Information |
| Completeness of External Information |
| Workflow - Integration of Vista Web and external information into clinical practice |
| Usability- Usefulness of VistaWeb, How information is displayed in VistaWeb |

- What concerns did your facility initially have with VLER implementation that proved to be non-issues?
- Based on your experience implementing VLER what advice would you give to another VA facility to make their implementation easier or better

***ALL PARTICIPANTS***

How well was VLER incorporated into the clinical workflow?

Why do you say that?

How would you describe the following characteristics of VLER data, on a scale of 1-10.

- Reliability
- Completeness
- Timeliness
- Overall quality
- Probe: why did you give the scores you did? Ask this after each rating is given. If not a 10 (best) then what would it take to make you give a higher rating in this area?

Did local champions of the VLER program emerge? Who were they and why do they advocate for the VLER program in your facility?

What is your organization's satisfaction with participation in VLER?

Would you modify anything about VLER? If so, what?

Would you recommend other VA facilities participate in VLER? Why/Why not?

What advice would you give to the central office (specify office name here?) as they continue to deploy VLER to other VA facilities like yours?

What role do you see VLER playing in your facility in 5 years? 10 years?

How did your facility plan to sustain VLER activity in the future?

Probes:

- When central VA business office remuneration funds are exhausted, does you facility plan to pay for continued exchange activity with regional HIEs?
- Do you believe that your regional HIE partners will be sustainable in the future, enabling VLER to continue?
- Do you believe the regional HIE partner(s) will exchange data with VA without the presence of remuneration funds?
- What would it take to sustain current VLER activities in the future without funding from central VA?
