# Supplementary material for: Leadership Perspectives on Implementing Health Information Exchange: Qualitative Study in a Tertiary Veterans Affairs Medical Center
Source: JMIR Med Inform. 2021 Feb 22;9(2):e19249. doi: 10.2196/19249 (PMC7939932; doi:10.2196/19249)
Supplement: Multimedia Appendix 3 [file medinform_v9i2e19249_app3.docx]

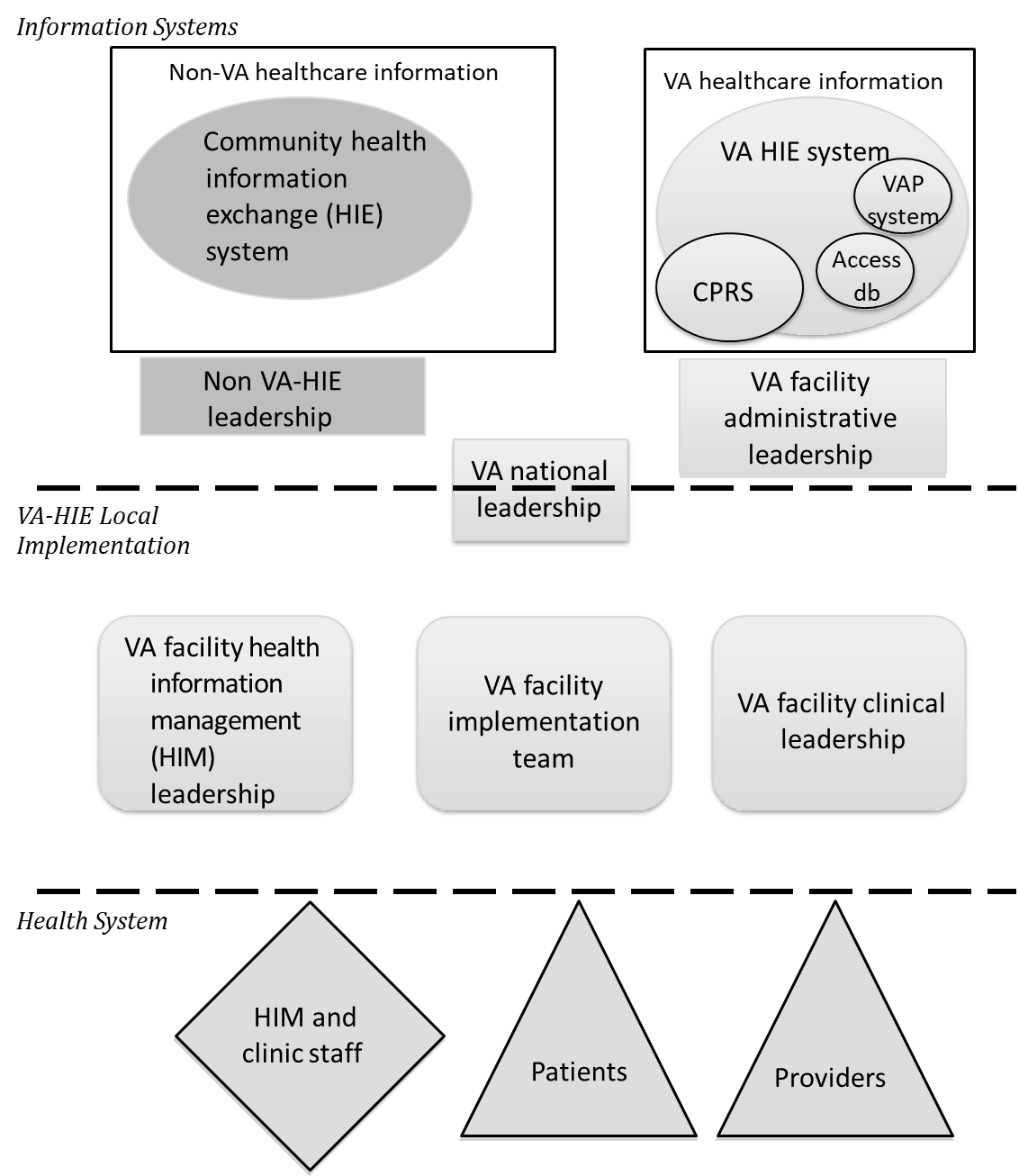


Figure 1. Groups and systems important to Department of Veterans Affairs-health information exchange integration at one VA medical center.

Our framework (Figure 1) represents the key stakeholder groups, and the relationships among them, necessary to integrate two complex health information systems. The model divides the data systems and stakeholder groups into three levels. At the top layer of the figure rests the information systems. Electronic medical information available about patients both inside and outside the VA health care system is represented in this layer. The middle layer consists of the organizational leadership and teams assembled in order to implement the HIE data systems in the health care setting. Finally, the bottom layer consists of key stakeholders, whether patient, providers, or other hospital staff who may not have a direct role in implementation of the information system but whose work is impacted by HIE.

Information Systems
Within the *Information Systems* layer*,* VA and non-VA health care information represent two key categories of data. The HIE and VA electronic information systems, represented by circles, indicate the technologies that contain patient data. Some medical records are not represented within the HIE or VA systems, hence the circle within the square demonstrates that neither system is fully complete.

Multiple sub-systems relevant to the demonstration project reside within the VA system: CPRS (Computerized Patient Record System), Access database used as a local registry to track enrolled Veterans, and VAP (Veteran Authorization Preference) the national registry of enrolled Veterans. CPRS is the existing enterprise EHR system of the VA and is the primary pathway for care providers to view patient information.

The Access database, shown here as a subset of the VA system, is a local registry that tracks consent of veterans who opt in to VA-HIE, and the expiration date of their consent. The VAP system contains the consent records of veterans and provides the necessary documentation of permission for HIE between VA and non-VA information systems at the national level.

VA Administrative Leadership and non-VA-HIE Leadership are charged with responsibility for the performance and governance of their respective information systems. Leadership groups of both the VA and community HIE partner included high-level support from the positions of CEO and CIO. Finally, the VA National Leadership in VHA central office shared expertise in the deployment and adaptation of HIE systems technology with both the VA and non-VA-HIE community partners. They also consulted with partners at the local demonstration site, thereby acting as a facilitator between information system and implementation groups.

VA-HIE Implementation
The *VA-HIE Implementation* level of activity illustrates the VA groups active in conducting HIE implementation. The VA Facility Implementation team had the largest role. This team included the Chief Health Informatics Officer, Community Coordinator (reporting to VA National Leadership) as well as of programmers and technicians who maintained and provided trouble-shooting for the technology interface between the VA and community HIE. VA facility HIM leadership provided protocols and procedures for obtaining consent, as well as advertising the availability of the VA HIE system to veterans seeking care at the local facility. Facility Clinical Leadership had less direct involvement in implementation, but supervised how the VA HIE system was incorporated into clinical workflow for health care providers.

Health System
The bottom level of interaction, encompassed within the *Health System*, visualizes the types of individuals with functional roles in the clinic. HIM and Clinic staff were the individuals who made face-to-face contact with veterans to obtain consent. They also explained how HIE information could be accessed to other clinical staff and providers less familiar with the VA HIE system. Providers deliver in-person care to veterans, including physicians, nurses, and medical assistants. Finally, patients are impacted by the VA-HIE system within the context of the patient-provider relationship. Patients can access VA-HIE information through the VA patient portal My-Health-eVet.

# Legend

VA: US Department of Veterans Affairs

HIE: Health Information Exchange

VAP: Veteran Authorization Preference registry (national)

CPRS: Computerized Patient Record System

Access db: Database / registry of veteran enrollees (local)

HIM: Health Information Management
